# Association between cognitive impairment and motor dysfunction among patients with multiple sclerosis: a cross-sectional study

**DOI:** 10.1186/s40001-023-01079-6

**Published:** 2023-03-02

**Authors:** Hanadi Matar Alharthi, Muneera Mohammed Almurdi

**Affiliations:** Rehabilitation of Health Sciences Department, College of Applied Medical Sciences, King Saud University, Riyadh, Saudi Arabia

**Keywords:** Multiple sclerosis, Motor dysfunction, Cognitive impairment, Risk of fall

## Abstract

**Background:**

Previous studies have shown that there is a relationship between cognitive impairment (CI) and motor dysfunction (MD) in neurological diseases, such as Alzheimer’s and Parkinson’s disease. However, there whether CI and MD are associated in patients with multiple sclerosis (MS) is unknown. Here we studied the association between CI and MD in patients with MS and examined if muscle weakness or incoordination, balance impairment, gait abnormalities, and/or increased fall risk are indicators of CI in patients with MS.

**Methods:**

Seventy patients with MS were included in this cross-sectional study. Cognitive impairment was assessed using the Montreal Cognitive Assessment Scale (MoCA), muscle strength using a hand-held dynamometer, and balance, gait, and fall risk assessment using the Tinetti scale. Motor coordination was assessed using the timed rapid alternating movement test for the upper extremity and the timed alternate heel-to-knee test for the lower extremity.

**Results:**

There was a significant association between CI and motor coordination, balance, gait, and risk of fall (*p* < 0.005) but not muscle strength. Stepwise multiple linear regression showed that 22.7% of the variance in the MoCA was predicted by the fall risk and incoordination of the upper extremities in the MS population.

**Conclusions:**

CI is significantly associated with motor incoordination, balance impairment, gait abnormality, and increased fall risk. Furthermore, the risk of fall and upper extremity incoordination appeared to be best indicators of CI in patients with MS.

**Supplementary Information:**

The online version contains supplementary material available at 10.1186/s40001-023-01079-6.

## Background

Multiple sclerosis (MS) is an inflammatory immune-mediated disease of the central nervous system (CNS) [1–3]. MS affects about 2.8 million people worldwide [4], and in 2018 its incidence was 40.4/100,000 of the population in the Kingdom of Saudi Arabia (KSA) [5]. It is more common in young adults aged 20–40 years of age [6] and in women than men (ratio 2:1) around the world, including in Saudi Arabia [4].

The most common clinical manifestations of MS are numbness, urinary/bowel symptoms, ataxia, tremor or dysmetria, visual disturbance, cognitive impairment (CI), and motor dysfunction (MD) [3, 7, 8]. Motor and cognitive dysfunction are the most common complications in patients with MS that negatively impact their quality of life (QoL) [9–11].

The MD seen in MS can take several forms including mobility problems, muscle weakness, spasticity, motor incoordination, and balance dysfunction [8, 12]. About 41% of patients with MS have walking difficulties that increase the fall risk and affect their daily living [13–15].

CI is also common in patients with MS, with a reported prevalence ranging between 43% and 70% [16–18]. It varies in severity and presents in all types of MS [19]. The most affected domains of CI are executive dysfunction, lack of attention, loss of memory, and slow information processing [20–22]. CI may disturb social interactions, work participation [23], and daily living in patients with MS [15, 24].

Understanding the relationship between motor and cognitive impairments is crucial to designing appropriate treatment plans that consider the patient’s unique symptom profile and that provides holistic care relevant to the success of physical therapy. In general, physical therapists do not give adequate attention to cognitive assessment in clinical practice. However, practitioners must be aware that the clinical assessment of cognitive function is no less important than a physical assessment of patients with MS, because it helps to determine the site of impairment and its relationship with physical abilities [21, 25, 26].

Previous studies have shown that there is a relationship between MD and CI in other neurological diseases, such as Alzheimer’s and Parkinson’s disease [27–29]. To date, similar previous studies on multiple sclerosis have focused on examining the relationship between motor and cognitive deficits [10, 21, 25, 30–34] and its negative effect on the overall functioning of MS adults [15]. Moreover, a retrospective study has revealed a significant correlation between cognitive function and a decrease in walking speed and an increase in the frequency of falls in patients with MS. However, these findings about walking speed were documented by nurses in the clinic visit and also the data of the repetition of falls were self-reported by patients or/and their family members based on their memory of the recent falls [34]. In addition, an observational pilot study reported that recurrent fallers have higher CI compared to single-time fallers among elderly people with MS [22]. While most studies focus on cognitive impairment as a predictor of the risk of fall, walking speed [34], gait variability [35], and postural control [32], it remains unclear whether the muscle strength, motor coordination, balance, gait, and/or risk of fall can be considered as predictors of CI in adults with MS.

Therefore, the aims of this study were to: (1) investigate the association between CI and muscle weakness, motor incoordination, poor balance, gait abnormality, and high fall risk in patients with MS; and (2) examine if muscle weakness, motor incoordination, poor balance, gait abnormalities, and/or increased fall risk can be adopted as the best indicator of CI in patients with MS.

## Methods

### Participants and inclusion and exclusion criteria

Seventy patients with MS were recruited for this cross-sectional study over 6-month period from July 2020 to January 2021. The sample size was estimated based on the G*Power3 analysis program when the effect size = 0.15, α error probability = 0.05, power (1-β error probability) = 0.8, and the number of predictors = 3 [36] which is reported according to STROBE guidelines [37] (Additional file 1). Patients were assessed in the Rehabilitation Department of King Khalid University Hospital, Physical Therapy Clinics of King Saud University and Sultan Bin Abdulaziz Humanitarian City. All measurements were conducted by a trained physical therapist with over 12 years of experience in rehabilitation of MS. Adults aged between 20 and 60, with a diagnosis of MS, able to read and write, and able to walk with or without assistive aids were included. Patients with communication difficulties, severe muscle weakness and spasticity, vision and hearing problems, severe CI, relapse within the last 3 months, neurological diseases other than MS, and/or a history of psychiatric illness were excluded.

### Instruments and procedures

#### Montreal Cognitive Assessment (MoCA)

The MoCA is a brief test that is used to assess cognitive function with a universal cutoff point of 26 to detect CI [38, 39]. The MoCA assesses several cognitive domains: (i) executive function, which involves an alternating trial-making task, a verbal fluency task, and a verbal abstracting task; (ii) the short-term memory domain, consisting of two trials of five words, learning and recalling them after about 5 min; (iii) the visuospatial domain, which includes drawing a clock and copying a cube; (iv) the language domain, covering naming, repetition of two sentences, and verbal fluency; (v) the attention assessment, which involves reading a list of digits forwards and backwards and serial subtraction; and the (vi) vigilance and (vii) abstraction domains as well as (viii) orientation to time and place [38]. In this study, the Arabic version of the MoCA was used by an examiner trained and certified in the MoCA to assess cognitive function [40]. The final score was the total of all correct points, and an additional point was given if the participant’s educational level was equal to or less than 12 years [38].

#### Muscle strength: handheld dynamometers

Isometric muscle strength was measured using “Jamar hydraulic hand dynamometer (Lafayette Instruments, Lafayette, Indiana, USA)” [41] for hand grip and “hand-held dynamometer (Commander Muscle Tester, JTech, USA)” [42] for knee extensor muscles. Three readings were recorded, and the highest value was selected, as previously [43]. For handgrip strength, the participant was in a sitting position and was asked to squeeze the dynamometer with the shoulder adducted and the elbow flexed 90°. For knee extensors, the participant was in a sitting position with the knee in 90° flexion. The examiner held the handheld dynamometer on the anterior lower third of the participant’s leg during isometric knee extension.

#### Lower extremity coordination test: timed alternate heel-to-knee test

In an alternative heel-to-knee test, the participant was in a supine position and was asked to bend the knee and drag the heel of the tested leg to reach the level of the contralateral knee, then extend the tested knee completely. The examiner used a stopwatch to time in seconds how long it took to finish ten repetitions for each side as quickly and accurately as possible [44, 45]. Two trials were performed for each side, and the faster one was selected [46].

#### Upper extremity coordination test: timed rapid alternating movement

The participant was in a sitting position and was asked to perform the test for each side separately after verbal instructions and a visual demonstration performed by the examiner. The participant was asked to perform alternate supination–pronation movements ten times as quickly and accurately as possible. The examiner used a stopwatch and counted the time in seconds from the beginning until the end of ten repetitions. The participant was asked to perform two trials, and the faster one was selected [46].

#### Tinetti Performance Oriented Mobility Assessment (POMA)

The POMA is a performance-based test to assess both balance (POMA-B) and gait (POMA-G) separately, and the total score indicates the fall risk [47, 48]. The POMA consists of nine tasks for balance assessment and seven tasks for gait assessment, with each task given a score of 0, 1, or 2, where 0 implies a low independence level and 2 a normal independence level [48]. The fall risk is considered high when the total score is ≤ 18, moderate with a score between 19 and 23, and low when it is ≥ 24 [48]. For balance assessment, the subject sat on a hard, armless chair and followed the examiner’s instructions. The assessment included sitting balance, rising, attempts to rise, immediate standing balance within the first five seconds, standing balance, nudged, eyes closed while standing, turning 360°, and sitting down [48]. For gait assessment, the subject had to walk 15 feet at their usual speed and back at safe rapid speed; participants could use an assistive device in this task. The assessment included gait initiation, step length and height, step symmetry, step continuity, path, trunk sway, and walking distance [47].

### Statistical analysis

All statistical analyses were performed using SPSS (v23, IBM Statistics, Armonk, NY). Participant characteristics were presented by descriptive analysis. A normality test was conducted on all variables to select the appropriate (parametric or non-parametric) statistical test. Pearson’s correlation coefficient analysis was used to assess the strength and direction of the association between cognition, muscle strength of knee extension, and motor coordination of upper extremity variables. The Spearman correlation coefficient was computed to assess associations between cognition, muscle strength of the hand grip, motor coordination of lower extremity balance, gait, and risk of fall. In addition, the Spearman correlation coefficient was used to assess the correlation between the MoCA domains and all motor variables. Motor variables significantly correlated with the MoCA were used in a multiple linear regression to determine the predictor variables [33, 49]. All data were expressed as mean ± standard deviation (SD), mode, median, or frequency and percentage, as required for each data type. The significance level was a *p* value ˂0.05 with a 95% confidence interval (CI).

## Results

### Participants and demographics

Seventy subjects were enrolled, 55 females and 15 males, with a mean age of 36.60 ± 9.24 years. The participants’ demographic characteristics are presented in Table 1.

**Table 1:** Participant demographics

Characteristic	Value [mean ± SD or n (%)] (*n* = 70)
Age (years) (mean ± SD)	36.60 ± 9.24
Handedness (mode)	1.00
Right [*n* (%)]	62 (88.6)
Gender (mode)	1.00
Female [*n* (%)]	55 (78.6)
MS subtype	
RRMS [*n* (%)]	65 (92.9)
SPMS [*n* (%)]	2 (2.9)
PPMS [*n* (%)]	3 (4.3)
Age at onset of symptoms (years) (mean ± SD)	26.11 ± 8.162
Disease duration (years) (mean ± SD)	10.60 ± 7.198
BMI (kg/m^2)^ (mean)	26.06 ± 5.49
Education (years) (mean ± SD)	14.84 ± 2.62

*SD* standard deviation, *RRMS* relapsing–remitting multiple sclerosis, *SPMS* secondary progressive multiple sclerosis, *PPMS* primary progressive multiple sclerosis, *BMI* body mass index

### Cognitive and motor variables

All cognitive function and motor variable data are summarized in Table 2. Memory was the most affected domain (in 84.3% of cases), followed by executive functions and visuospatial skills at 74% and 71.4%, respectively. Language was only affected in 26% of cases. The worst score from both sides was selected for all motor variables examined bilaterally.

**Table 2:** Descriptive analysis of the cognitive and motor variables

Parameter	Value [mean ± SD or n (%)] (*n* = 70)
MoCA (total) (mean ± SD)	23.51 ± 3.55
MoCA level (%)	
Normal (26–30)	19 (27.1)
Mild CI (18–25)	47 (67.1)
Moderate CI (10–17)	4 (5.7)
MoCA domains (%)	
Executive functions	74
Visuospatial skills	71.4
Memory	84.3
Attention	65.7
Language	26
Strength of upper extremities	
Handgrip strength of dominant hand (kg) (mean ± SD)	23.41 ± 6.65
Handgrip strength of non-dominant hand (kg) (mean ± SD)	22.74 ± 7.44
Strength of lower extremities	
Rt-knee extensors (quadriceps) strength (kg) (mean ± SD)	26.91 ± 8.52
Lt-knee extensors (quadriceps) strength (kg) (mean ± SD)	26.24 ± 8.22
Motor coordination of upper extremities	
Supination/pronation of dominant hand (sec) (mean ± SD)	06.93 ± 01.74
Supination/pronation of non-dominant hand (sec) (mean ± SD)	07.11 ± 01.75
Motor coordination of lower extremities	
Rt-heel-to-knee (sec) (mean ± SD)	17.15 ± 06.66
Lt-heel-to-knee (sec) (mean ± SD)	15.49 ± 05.89
POMA-B total score (mean ± SD)	13.00 ± 3.67
POMA-G total score (mean ± SD)	8.76 ± 3.26
Overall POMA score (mean ± SD)	21.81 ± 6.66
Low fall risk [*n* (%)]	40 (57.1)
Moderate fall risk [*n* (%)]	11 (15.7)
High fall risk [*n* (%)]	19 (27.1)

*MoCA* Montreal Cognitive Assessment Scale, *Rt* right side, *Lt* left side, *POMA-B* balance component of POMA, *POMA-G* gait component of POMA, *SD* standard deviation

### Correlation between cognitive performance (MoCA) and motor function

As highlighted in Table 3, the correlation analysis was computed between Motor variables including muscle strength of handgrip and knee extension, motor coordination of upper and lower extremities, balance, gait, and the risk of fall.

**Table 3:** Correlations analysis between cognitive and motor scores

Cognitive functions variables	Motor variables						
	Muscle strength		Motor coordination		POMA (Tinetti scale)		
	Handgrip	Knee Extension	Supination/Pronation	Heel to Knee	Balance	Gait	Risk of Fall
MoCA (Total Score)	0.179	0.146	−0.412**	−0.260*	0.445**	0.440**	0.468**
Attention	0.314**	0.148	− 0.080	−0.017	0.201	0.222	0.233
Language	0.434**	−0.033	−0.296*	−0.308**	0.318**	0.272*	0.316**
Orientation	0.022	−0.015	−0.121	−0.079	0.325**	0.248*	0.277*
Memory	−0.110	0.056	−0.280*	−0.129	0.318**	0.300**	0.307**
Visuospatial Skills	0.136	0.096	−0.278*	−0.138	0.312**	0.293*	0.319**
Executive Functions	0.034	0.034	−0.287*	−0.234	0.262*	0.214	0.242*

*POMA*  Performance Oriented Mobility Assessment

**Correlation significant at *p* < 0.01

*Correlation significant at *p* < 0.05

### Motor predictor variables

**Fig. 1:**
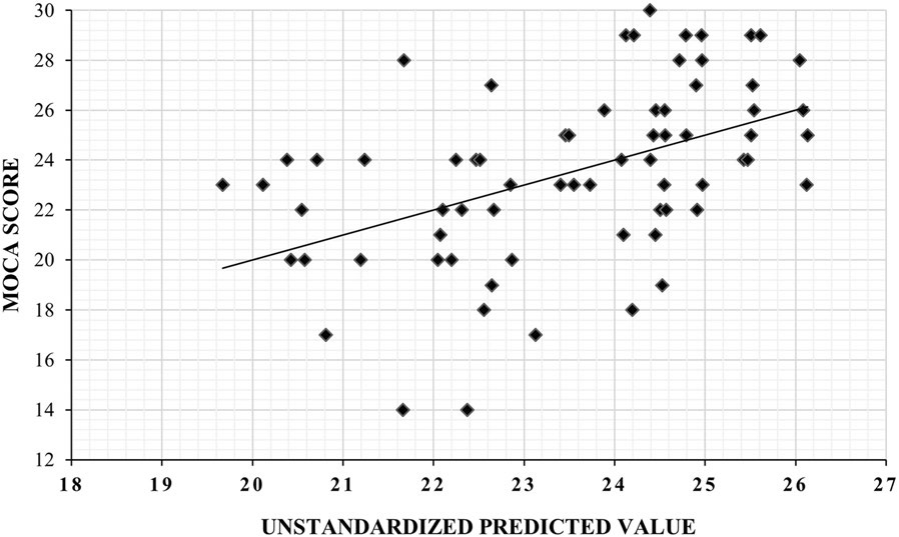
Scatter plot of the MoCA on the overall POMA score (fall risk) and upper extremity coordination score with regression line and individual 95% CIs

Motor variables significantly correlated with the MoCA were used in a multiple linear regression analysis to determine predictor variables [50, 51]. Balance (POMA-B) and gait (POMA-G) were excluded from the linear regression, because they were highly correlated with each other (*ρ* = 0.822, *n* = 70, *p* ˂ 0.001), balance (POMA-B) with fall risk (POMA total score) (*ρ* = 0.931, *n* = 70, *p* ˂ 0.001), and gait (POMA-G) with fall risk (POMA total score) (*ρ* = 0.961, *n* = 70, *p* ˂ 0.001). Strength of hand grip and knee extension were also excluded, because they were not significantly correlated with the MoCA. Using the total POMA score (fall risk), upper and lower limb coordination variables were entered into the regression analysis as independent variables and the MoCA as the dependent variable. Using the stepwise method, a significant model 1 emerged (F (1, 68) = 14.150, *p* < 0.001) with an *R*^2^ of 0.172 (Table 4). Therefore, 17.2% of the variance in the MoCA was predictable from the overall POMA score (fall risk). In model 2 (F (2, 67) = 9.814, *p* < 0.001), the *R*^2^ increased from 0.171 to 0.227 after adding the supination/pronation test (Tables 4 and 5). Thus, 22.7% of the variance in the MoCA was predictable from the overall POMA score (fall risk) and supination/pronation test (Fig. 1). The equation describing the best fit of the regression is

**Table 4:** Summary of multiple linear regression analysis: dependent variable–MoCA total score

Model	*R*	*R*^2^	Adjusted *R*^2^	SE	*F*	*P*
1	0.415^a^	0.172	0.160	3.250	14.150	< 0.001
2	0.476^b^	0.227	0.203	3.164	9.814	< 0.001

*R* Correlation coefficient, *R*^2^ Coefficient of determination, *SE* Standard error.

^a^Predictors: Risk of Fall

^b^Predictors: (Constant), Risk of Fall, Supination/Pronation

**Table 5:** Summary of stepwise multiple linear regression analysis for predicting cognitive impairment (MoCA score)

Model	Variables	*B*	SE	*β*
1	Constant	18.698	1.338	
	Fall Risk	0.221	0.059	0.415**
2	Constant	24.325	2.903	
	Fall Risk	0.147	0.066	0.277*
	Supination/Pronation	−0.539	0.248	−0.271*

*B* Unstandardized beta coefficient; *SE* Standard error; *β*  Standardized beta coefficient.

*P < 0.05.

*P < 0.001

$$MoCA \, = \, 24.325 +(0.147 \times \left(Fall Risk\right)+(-0.539 \times \left(Supination\setminus Pronation\right))$$

A one-unit increase in the total POMA score increased the MoCA score by 0.147, which was significant (*t* (67) = 2.216, *p* < 0.001) (Table 5). In addition, a one-unit increase in the supination/pronation score decreased the MoCA score by 0.539, which was also significant (*t* (67) =−2.169, *p* < 0.001).

## Discussion

The results of this study showed that CI is significantly associated with motor incoordination of the upper and lower extremities, balance deficits, gait abnormalities, and fall risk. However, there was no significant association between cognition and muscle strength, as estimated by hand grip and knee extension strength. Moreover, reduced upper and lower extremity coordination scores and lower fall risk were associated with higher CI scores. Specifically, the fall risk and upper extremity incoordination scores were predictive of CI in MS patients.

The results confirmed that the mean total MoCA score indicated mild cognitive impairment and in about two thirds of patients with CI, consistent with previous studies [52, 53]. Moreover, several studies have shown that the most affected cognitive domains in MS patients were memory, abstract/conceptual reasoning, information processing, attention, and visuospatial skills [26, 54]. Similarly, we found that memory, visuospatial skills, executive function, attention, and language were affected in patients with CI based on the MoCA scale.

This study found no association between isometric knee extensor strength and CI. Conversely, a recent study reported a significant correlation between CI and knee extension strength in MS patients [33]. Sandroff et al. [55] reported that the peak torque of knee extension was associated with cognitive processing speed but not verbal and visuospatial learning and memory in 62 people with MS. These contradictory results might be due smaller sample sizes or the type of dynamometer used. Similar to other studies, there was no correlation between hand grip strength and CI as measured by the total MoCA score [33], but there was a significant correlation between hand grip strength and the language and attention domains.

The current study revealed significant correlations between motor incoordination of the upper and lower extremities and CI in patients with MS. Poor performance on rapid alternating movement and heel-to-knee tests is usually due to cerebellar dysfunction [56]. Furthermore, several studies have shown that the cerebellum plays an important role in both the cognitive and motor functions of MS patients [56–58]. This might be because the cerebello-cerebral network, consisting of the forward cortico-ponto-cerebellar pathway and the backward cerebello-thalamo-cortical pathway, also modulates cognition [59, 60].

A balance component of POMA was significantly moderately correlated with cognitive performance in MS patients. This is the first study examining the association between cognition and balance in patients with MS using the POMA-B scale. Alfonso et al. [61] similarly concluded that cognitive impairment in adults with MS including processing speed deficit was associated with difficulty selecting appropriate corrective movements to compensate for balance disturbances. This speculation is consistent with a previous study that found postural control becomes more difficult in MS individuals due to somatosensory and visual input deficits. Consequently, additional compensatory mechanisms are needed to overcome this postural instability which also requires the support of several cognitive domains [62]. Moreover, other studies have examined postural–cognitive interference by incorporating the dual-task paradigms in healthy people and those with MS [63, 64]. A recent review concluded that patients with MS showed impaired balance when they simultaneously performed a cognitive and postural task, which increased their fall risk in most daily activities [63].

The gait component of the POMA was moderately positively correlated with cognition, consistent with previous studies demonstrating a significant association between gait abnormalities and cognitive decline in people with MS [31, 35]. A previous study reported that step length and step time variabilities were associated with cognitive processing speed in MS patients [31]. It is interesting to note that no study used POMA-G for the assessment of gait and its correlation with cognition in MS individuals. The current study is, therefore, unique in its use of a simple, short, inexpensive scale that does not need specialist equipment in clinical practice.

Fall risk was significantly correlated with cognitive impairment and predicted CI in MS individuals, consistent with previous studies [34, 65]. Moreover, a higher POMA score was associated with a decrease in fall risk, which was associated with higher MoCA scores. This is particularly important when investigating the fall risk during motor examination in patients with MS and provides a clue about the presence of cognitive impairment. Furthermore, fall frequency has been shown to be significantly correlated with general intelligence, speed of cognitive processing [22], and executive functioning, while verbal memory was found to be a significant predictor of falls in patients with MS [34].

This study has several limitations. The sample size was small, and further studies are required with a larger sample size to improve the robustness of regression. Most of the participants had relapsing–remitting MS which limiting the generalizability of the results on the whole MS population and future studies should involve all MS subtypes. The POMA-G used in this study is based on the examiner’s observations and is less sensitive for detecting gait abnormalities than other instruments. Therefore, future studies using advanced gait analysis systems are now needed to better detect temporal and visuospatial parameters.

## Conclusions

CI is significantly associated with motor incoordination, poor balance, gait abnormalities, and increased fall risk. The fall risk and upper extremity incoordination were the best indicators of CI in patients with MS. Thus, motor assessment can provide physical therapists with clues about the presence of CI in patients with MS. In addition, incorporating coordination and balance training into the rehabilitation program may enhance cognitive functions in patients with MS, although this requires empirical testing.

## Supplementary Information

**Additional file: 1.** STROBE STATEMENT-checklist

## Data Availability

The data set supporting the conclusions of this article is included within the article (and its additional file).

## Abbreviations

BMI: Body mass index
CNS: Central nervous system
CI: Cognitive impairment
CI: Confidence interval
KSA: Kingdom of Saudi Arabia
MoCA: Montreal Cognitive Assessment
MD: Motor dysfunction
MS: Multiple sclerosis
PPMS: Primary progressive multiple sclerosis
QoL: Quality of life
RRMS: Relapsing–remitting multiple sclerosis
SPMS: Secondary progressive multiple sclerosis
SD: Standard deviation
POMA: Tinetti Performance Oriented Mobility Assessment

## References

[1] Dobson R, Giovannoni G (2019). Multiple sclerosis—a review. Eur J Neurol.

[2] Frohman EM, Racke MK, Raine CS (2006). Multiple sclerosis–the plaque and its pathogenesis. N Engl J Med.

[3] Gelfand JM (2014). Multiple sclerosis: diagnosis, differential diagnosis, and clinical presentation. Handb Clin Neurol.

[4] Walton C, King R, Rechtman L, Kaye W, Leray E, Marrie RA (2020). Rising prevalence of multiple sclerosis worldwide: insights from the Atlas of MS third edition. Mult Scler.

[5] AlJumah M, Bunyan R, Al Otaibi H, Al Towaijri G, Karim A, Al Malik Y (2020). Rising prevalence of multiple sclerosis in Saudi Arabia, a descriptive study. BMC Neurol.

[6] Koch-Henriksen N, Sorensen PS (2010). The changing demographic pattern of multiple sclerosis epidemiology. Lancet Neurol.

[7] Kurtzke JF (1983). Rating neurologic impairment in multiple sclerosis: an expanded disability status scale (EDSS). Neurology.

[8] Huang WJ, Chen WW, Zhang X (2017). Multiple sclerosis: pathology, diagnosis and treatments. Exp Ther Med.

[9] Amato MP, Prestipino E, Bellinvia A (2019). Identifying risk factors for cognitive issues in multiple sclerosis. Expert Rev Neurother.

[10] Yozbatiran N, Baskurt F, Baskurt Z, Ozakbas S, Idiman E (2006). Motor assessment of upper extremity function and its relation with fatigue, cognitive function and quality of life in multiple sclerosis patients. J Neurol Sci.

[11] Bogosian A, Moss-Morris R, Yardley L, Dennison L (2009). Experiences of partners of people in the early stages of multiple sclerosis. Mult Scler.

[12] Schapiro RT (1994). Symptom management in multiple sclerosis. Ann Neurol.

[13] Finlayson ML, Peterson EW, Cho CC (2006). Risk factors for falling among people aged 45 to 90 years with multiple sclerosis. Arch Phys Med Rehabil.

[14] Larocca NG (2011). Impact of walking impairment in multiple sclerosis: perspectives of patients and care partners. Patient.

[15] Slavkovic S, Golubovic S, Vojnovic M, Nadj C (2019). Influence of cognitive and motor abilities on the level of current functioning in people with multiple sclerosis. Zdr Varst.

[16] Rao SM, Leo GJ, Bernardin L, Unverzagt F (1991). Cognitive dysfunction in multiple sclerosis. I. Frequency, patterns, and prediction. Neurology.

[17] Gaetani L, Salvadori N, Chipi E, Gentili L, Borrelli A, Parnetti L (2021). Cognitive impairment in multiple sclerosis: lessons from cerebrospinal fluid biomarkers. Neural Regen Res.

[18] Artemiadis A, Anagnostouli M, Zalonis I, Chairopoulos K, Triantafyllou N (2018). Structural MRI correlates of cognitive function in multiple sclerosis. Mult Scler Relat Disord.

[19] Bagert B, Camplair P, Bourdette D (2002). Cognitive dysfunction in multiple sclerosis: natural history, pathophysiology and management. CNS Drugs.

[20] Chiaravalloti ND, DeLuca J (2008). Cognitive impairment in multiple sclerosis. Lancet Neurol.

[21] Ozkul C, Guclu-Gunduz A, Eldemir K, Apaydin Y, Yazici G, Irkec C (2020). Clinical features and physical performance in multiple sclerosis patients with and without cognitive impairment: a cross-sectional study. Int J Rehabil Res Internationale Zeitschrift fur Rehabilitationsforschung Revue internationale de recherches de readaptation.

[22] Sosnoff JJ, Balantrapu S, Pilutti LA, Sandroff BM, Morrison S, Motl RW (2013). Cognitive processing speed is related to fall frequency in older adults with multiple sclerosis. Arch Phys Med Rehabil.

[23] Rao SM, Leo GJ, Ellington L, Nauertz T, Bernardin L, Unverzagt F (1991). Cognitive dysfunction in multiple sclerosis. II. Impact on employment and social functioning. Neurology.

[24] Schiavolin S, Leonardi M, Giovannetti AM, Antozzi C, Brambilla L, Confalonieri P (2013). Factors related to difficulties with employment in patients with multiple sclerosis: a review of 2002–2011 literature. Int J Rehabil Res Internationale Zeitschrift fur Rehabilitationsforschung Revue internationale de recherches de readaptation.

[25] Benedict RH, Holtzer R, Motl RW, Foley FW, Kaur S, Hojnacki D (2011). Upper and lower extremity motor function and cognitive impairment in multiple sclerosis. J Int Neuropsychol Soc.

[26] Macias Islas MA, Ciampi E (2019). Assessment and impact of cognitive impairment in multiple sclerosis: an overview. Biomedicines.

[27] Franssen EH, Souren LE, Torossian CL, Reisberg B (1999). Equilibrium and limb coordination in mild cognitive impairment and mild Alzheimer's disease. J Am Geriatr Soc.

[28] Aggarwal NT, Wilson RS, Beck TL, Bienias JL, Bennett DA (2006). Motor dysfunction in mild cognitive impairment and the risk of incident Alzheimer disease. Arch Neurol.

[29] Wang YX, Zhao J, Li DK, Peng F, Wang Y, Yang K (2017). Associations between cognitive impairment and motor dysfunction in Parkinson's disease. Brain Behav.

[30] Kalron A (2014). The relationship between specific cognitive domains, fear of falling, and falls in people with multiple sclerosis. Biomed Res Int.

[31] Hsieh KL, Sun R, Sosnoff JJ (2017). Cognition is associated with gait variability in individuals with multiple sclerosis. J Neural Transm (Vienna).

[32] Perrochon A, Holtzer R, Laidet M, Armand S, Assal F, Lalive PH (2017). Postural control is associated with cognition and fear of falling in patients with multiple sclerosis. J Neural Transm (Vienna).

[33] Aristotelous P, Stefanakis M, Pantzaris M, Pattichis C, Hadjigeorgiou GM, Giannaki CD (2019). Associations between functional capacity, isokinetic leg strength, sleep quality and cognitive function in multiple sclerosis patients: a cross-sectional study. Postgrad Med.

[34] D'Orio VL, Foley FW, Armentano F, Picone MA, Kim S, Holtzer R (2012). Cognitive and motor functioning in patients with multiple sclerosis: neuropsychological predictors of walking speed and falls. J Neurol Sci.

[35] Kalron A, Aloni R, Dolev M, Frid L, Givon U, Menascu S (2018). The relationship between gait variability and cognitive functions differs between fallers and non-fallers in MS. J Neural Transm (Vienna).

[36] Faul F, Erdfelder E, Buchner A, Lang A-G (2009). Statistical power analyses using G*Power 3.1: Tests for correlation and regression analyses. Behav Res Methods.

[37] von Elm E, Altman DG, Egger M, Pocock SJ, Gotzsche PC, Vandenbroucke JP (2014). The Strengthening the Reporting of Observational Studies in Epidemiology (STROBE) statement: guidelines for reporting observational studies. Int J Surg.

[38] Charvet LE (2015). The Montreal Cognitive Assessment (MoCA) in multiple sclerosis: relation to clinical features. J Mult Scler.

[39] Dagenais E, Rouleau I, Demers M, Jobin C, Roger E, Chamelian L (2013). Value of the MoCA test as a screening instrument in multiple sclerosis. Can J Neurol Sci.

[40] Rahman TT, El Gaafary MM (2009). Montreal Cognitive Assessment Arabic version: reliability and validity prevalence of mild cognitive impairment among elderly attending geriatric clubs in Cairo. Geriatr Gerontol Int.

[41] Cuesta-Vargas A, Hilgenkamp T (2015). Reference values of grip strength measured with a jamar dynamometer in 1526 adults with intellectual disabilities and compared to adults without intellectual disability. PLoS ONE.

[42] Kim SG, Lee YS (2015). The intra- and inter-rater reliabilities of lower extremity muscle strength assessment of healthy adults using a hand held dynamometer. J Phys Ther Sci.

[43] Trosclair D, Bellar D, Judge LW, Smith J, Mazerat N, Brignac A (2011). Hand-grip strength as a predictor of muscular strength and endurance. J Strength Cond Res.

[44] Lanzino DJ, Rabinstein A, Kinlaw D, Hepburn SB, Ness BM, Olson KJ (2012). Coordination tests in persons with acute central nervous system pathology: assessment of interrater reliability and known-group validity. J Neurol Phys Ther.

[45] Pinheiro MdB, Menezes KKPd, Teixeira-Salmela LF (2014). Review of the psychometric properties of lower limb motor coordination tests. Fisioterapia em Movimento..

[46] Lanzino DJ, Conner MN, Goodman KA, Kremer KH, Petkus MT, Hollman JH (2012). Values for timed limb coordination tests in a sample of healthy older adults. Age Ageing.

[47] Hayes KW, Johnson ME (2003). Measures of adult general performance tests: The Berg Balance Scale, Dynamic Gait Index (DGI), Gait Velocity, Physical Performance Test (PPT), Timed Chair Stand Test, Timed Up and Go, and Tinetti Performance-Oriented Mobility Assessment (POMA). Arthritis Rheum.

[48] Tinetti ME (1986). Performance-oriented assessment of mobility problems in elderly patients. J Am Geriatr Soc.

[49] Gonzalez-Chica DA, Bastos JL, Duquia RP, Bonamigo RR, Martínez-Mesa J (2015). Test of association: which one is the most appropriate for my study?. An Bras Dermatol.

[50] Allison PD (1999). Multiple regression: a primer.

[51] Miller AJ (1984). Sélection of subsets of regression variables. J Roy Stat Soc.

[52] Rao SM, Leo GJ, Bernardin L, Unverzagt F (1991). Cognitive dysfunction in multiple sclerosis.: I. Frequency, patterns, and prediction. Neurology.

[53] McNicholas N, O'Connell K, Yap SM, Killeen RP, Hutchinson M, McGuigan C (2018). Cognitive dysfunction in early multiple sclerosis: a review. QJM.

[54] Rao SM (2004). Cognitive function in patients with multiple sclerosis: impairment and treatment. Int J MS Care.

[55] Sandroff BM, Pilutti LA, Benedict RH, Motl RW (2015). Association between physical fitness and cognitive function in multiple sclerosis: does disability status matter?. Neurorehabil Neural Repair.

[56] Sarica A, Cerasa A, Quattrone A (2015). The neurocognitive profile of the cerebellum in multiple sclerosis. Int J Mol Sci.

[57] D'Ambrosio A, Pagani E, Riccitelli GC, Colombo B, Rodegher M, Falini A (2017). Cerebellar contribution to motor and cognitive performance in multiple sclerosis: an MRI sub-regional volumetric analysis. Mult Scler.

[58] Valentino P, Cerasa A, Chiriaco C, Nistico R, Pirritano D, Gioia M (2009). Cognitive deficits in multiple sclerosis patients with cerebellar symptoms. Mult Scler.

[59] Tedesco AM, Chiricozzi FR, Clausi S, Lupo M, Molinari M, Leggio MG (2011). The cerebellar cognitive profile. Brain.

[60] Schmahmann JD (2004). Disorders of the cerebellum: ataxia, dysmetria of thought, and the cerebellar cognitive affective syndrome. J Neuropsychiatry Clin Neurosci.

[61] Fasano A, Plotnik M, Bove F, Berardelli A (2012). The neurobiology of falls. Neurol Sci.

[62] Huisinga JM, Yentes JM, Filipi ML, Stergiou N (2012). Postural control strategy during standing is altered in patients with multiple sclerosis. Neurosci Lett.

[63] Chamard Witkowski L, Mallet M, Belanger M, Marrero A, Handrigan G (2019). Cognitive-postural interference in multiple sclerosis. Front Neurol.

[64] Beste C, Muckschel M, Paucke M, Ziemssen T (2018). Dual-tasking in multiple sclerosis - implications for a cognitive screening instrument. Front Hum Neurosci.

[65] Amboni M, Barone P, Hausdorff JM (2013). Cognitive contributions to gait and falls: evidence and implications. Mov Disord.

