# Supplementary material for: Association between cognitive impairment and motor dysfunction among patients with multiple sclerosis: a cross-sectional study
Source: Eur J Med Res. 2023 Mar 2;28:110. doi: 10.1186/s40001-023-01079-6 (PMC9979523; doi:10.1186/s40001-023-01079-6)
Supplement: Supplementary file 1 — Additional file: 1. STROBE STATEMENT-checklist [file 40001_2023_1079_MOESM1_ESM.docx]

STROBE Statement—checklist of items that should be included in reports of observational studies

|  | Item No. | Recommendation | Page  No. | Relevant text from manuscript |
| --- | --- | --- | --- | --- |
| **Title and abstract** | 1 | (*a*) Indicate the study’s design with a commonly used term in the title or the abstract | 1 | Association Between Cognitive Impairment and Motor Dysfunction in Patients with Multiple Sclerosis: A Cross-sectional Study |
|  |  | (*b*) Provide in the abstract an informative and balanced summary of what was done and what was found | 1-2 | **Methods:** Seventy patients with MS were included in this cross-sectional study. Cognitive impairment was assessed using the Montreal Cognitive Assessment Scale (MoCA), muscle strength using a hand-held dynamometer, and balance, gait, and fall risk assessment using the Tinetti scale. Motor coordination was assessed using the timed rapid alternating movement test for the upper extremity and the timed alternate heel-to-knee test for the lower extremity.  **Results:** There was a significant association between CI and motor coordination, balance, gait, and risk of fall (p < 0.005) but not muscle strength. Stepwise multiple linear regression showed that 22.7% of the variance in the MoCA was predicted by the fall risk and incoordination of the upper extremities in the MS population. |
| Introduction | | | |  |
| Background/rationale | 2 | Explain the scientific background and rationale for the investigation being reported | 2-3 | Previous studies have shown that there is a relationship between MD and CI in other neurological diseases such as Alzheimer’s and Parkinson’s disease [21-23]. However, to our best knowledge, there has been no study addressing the association between CI and MD in patients with MS.  Therefore, the aims of this study were to: (1) investigate the association between CI and muscle weakness, motor incoordination, poor balance, gait abnormality, and high fall risk in patients with MS; and (2) examine if muscle weakness, motor incoordination, poor balance, gait abnormalities, and/or increased fall risk can be adopted as the best indicator of CI in patients with MS. |
| Objectives | 3 | State specific objectives, including any prespecified hypotheses | 3 | Therefore, the aims of this study were to: (1) investigate the association between CI and muscle weakness, motor incoordination, poor balance, gait abnormality, and high fall risk in patients with MS; and (2) examine if muscle weakness, motor incoordination, poor balance, gait abnormalities, and/or increased fall risk can be adopted as the best indicator of CI in patients with MS. |
| Methods | | | |  |
| Study design | 4 | Present key elements of study design early in the paper | 3-4 | Seventy patients with MS were recruited for this cross-sectional study, which is reported according to STROBE guidelines [24] (**Supplementary File**). Patients were assessed in the Rehabilitation Department of King Khalid University Hospital, Physical Therapy Clinics of King Saud University and Sultan Bin Abdulaziz Humanitarian City. Adults aged between 20 and 60, with a diagnosis of MS, able to read and write, and able to walk with or without assistive aids were included. Patients with communication difficulties, severe muscle weakness and spasticity, vision and hearing problems, severe CI, relapse within the last three months, neurological diseases other than MS, and/or a history of psychiatric illness were excluded. |
| Setting | 5 | Describe the setting, locations, and relevant dates, including periods of recruitment, exposure, follow-up, and data collection | 3-4 | Seventy patients with MS were recruited for this cross-sectional study, which is reported according to STROBE guidelines [24] (**Supplementary File**). Patients were assessed in the Rehabilitation Department of King Khalid University Hospital, Physical Therapy Clinics of King Saud University and Sultan Bin Abdulaziz Humanitarian City. Adults aged between 20 and 60, with a diagnosis of MS, able to read and write, and able to walk with or without assistive aids were included. Patients with communication difficulties, severe muscle weakness and spasticity, vision and hearing problems, severe CI, relapse within the last three months, neurological diseases other than MS, and/or a history of psychiatric illness were excluded. |
| Participants | 6 | (*a*) *Cohort study*—Give the eligibility criteria, and the sources and methods of selection of participants. Describe methods of follow-up  *Case-control study*—Give the eligibility criteria, and the sources and methods of case ascertainment and control selection. Give the rationale for the choice of cases and controls  *Cross-sectional study*—Give the eligibility criteria, and the sources and methods of selection of participants | 3-4 | Seventy patients with MS were recruited for this cross-sectional study, which is reported according to STROBE guidelines [24] (**Supplementary File**). Patients were assessed in the Rehabilitation Department of King Khalid University Hospital, Physical Therapy Clinics of King Saud University and Sultan Bin Abdulaziz Humanitarian City. Adults aged between 20 and 60, with a diagnosis of MS, able to read and write, and able to walk with or without assistive aids were included. Patients with communication difficulties, severe muscle weakness and spasticity, vision and hearing problems, severe CI, relapse within the last three months, neurological diseases other than MS, and/or a history of psychiatric illness were excluded. |
|  |  | (*b*) *Cohort study*—For matched studies, give matching criteria and number of exposed and unexposed  *Case-control study*—For matched studies, give matching criteria and the number of controls per case |  |  |
| Variables | 7 | Clearly define all outcomes, exposures, predictors, potential confounders, and effect modifiers. Give diagnostic criteria, if applicable | 6-10 | Motor variables significantly correlated with the MoCA were used in a multiple linear regression to determine the predictor variables.  Using the total POMA score (fall risk), upper and lower limb coordination variables were entered into the regression analysis as independent variables and the MoCA as the dependent variable. |
| Data sources/ measurement | 8* | For each variable of interest, give sources of data and details of methods of assessment (measurement). Describe comparability of assessment methods if there is more than one group | 4-6 | **Instruments and procedures**   - *Montreal Cognitive Assessment (MoCA)*   The MoCA is a brief test that is used to assess cognitive function with a universal cut-off point of 26 to detect CI [25, 26]. The MoCA assesses several cognitive domains: (i) executive function, which involves an alternating trial-making task, a verbal fluency task, and a verbal abstracting task; (ii) the short-term memory domain, consisting of two trials of five words, learning and recalling them after about five minutes; (iii) the visuospatial domain, which includes drawing a clock and copying a cube; (iv) the language domain, covering naming, repetition of two sentences, and verbal fluency; (v) the attention assessment, which involves reading a list of digits forwards and backwards and serial subtraction; and the (vi) vigilance and (vii) abstraction domains as well as (viii) orientation to time and place [25]. In this study, the Arabic version of the MoCA was used by an examiner trained and certified in the MoCA to assess cognitive function [27]. The final score was the total of all correct points, and an additional point was given if the participant’s educational level was equal to or less than 12 years [25].   - *Muscle strength: handheld dynamometers*   Isometric muscle strength was measured using a handheld dynamometer for hand grip and knee extensor muscles. Three readings were recorded, and the highest value was selected, as previously [28]. For handgrip strength, the participant was in a sitting position and was asked to squeeze the dynamometer with the shoulder adducted and the elbow flexed 90°. For knee extensors, the participant was in a sitting position with the knee in 90° flexion. The examiner held the handheld dynamometer on the anterior lower third of the participant’s leg during isometric knee extension.   - *Lower extremity coordination test: timed alternate heel-to-knee test*   In an alternative heel-to-knee test, the participant was in a supine position and was asked to bend the knee and drag the heel of the tested leg to reach the level of the contralateral knee, then extend the tested knee completely. The examiner used a stopwatch to time in seconds how long it took to finish ten repetitions for each side as quickly and accurately as possible [29, 30]. Two trials were performed for each side, and the faster one was selected [31].   - *Upper extremity coordination test: timed rapid alternating movement*   The participant was in a sitting position and was asked to perform the test for each side separately after verbal instructions and a visual demonstration performed by the examiner. The participant was asked to perform alternate supination-pronation movements ten times as quickly and accurately as possible. The examiner used a stopwatch and counted the time in seconds from the beginning until the end of ten repetitions. The participant was asked to perform two trials, and the faster one was selected [31].   - *Tinetti Performance Oriented Mobility Assessment (POMA)*   The POMA is a performance-based test to assess both balance (POMA-B) and gait (POMA-G) separately, and the total score indicates the fall risk [32, 33]. The POMA consists of nine tasks for balance assessment and seven tasks for gait assessment, with each task given a score of 0, 1, or 2, where 0 implies a low independence level and 2 a normal independence level [33]. The fall risk is considered high when the total score is ≤18, moderate with a score between 19 and 23, and low when it is ≥24 [33]. For balance assessment, the subject sat on a hard, armless chair and followed the examiner’s instructions. The assessment included sitting balance, rising, attempts to rise, immediate standing balance within the first five seconds, standing balance, nudged, eyes closed while standing, turning 360°, and sitting down [33]. For gait assessment, the subject had to walk 15 feet at their usual speed and back at safe rapid speed; participants could use an assistive device in this task. The assessment included gait initiation, step length and height, step symmetry, step continuity, path, trunk sway, and walking distance [32]. |
| Bias | 9 | Describe any efforts to address potential sources of bias | 4 | All measurements were conducted by a trained physical therapist with over 12 years of experience in rehabilitation of MS |
| Study size | 10 | Explain how the study size was arrived at | 3 | Seventy patients with MS were recruited for this cross-sectional study and the sample size was estimated based on the G*Power3 analysis program |

Continued on next page

| Quantitative variables | 11 | Explain how quantitative variables were handled in the analyses. If applicable, describe which groupings were chosen and why | 6 | All statistical analyses were performed using SPSS (v23, IBM Statistics, Armonk, NY). Participant characteristics were presented by descriptive analysis. A normality test was conducted on all variables to select the appropriate (parametric or non-parametric) statistical test. Pearson’s correlation coefficient analysis was used to assess the strength and direction of the association between cognition, muscle strength of knee extension, and motor coordination of upper extremity variables. The Spearman correlation coefficient was computed to assess associations between cognition, muscle strength of the hand grip, motor coordination of lower extremity balance, gait, and risk of fall. In addition, the Spearman correlation coefficient was used to assess the correlation between the MoCA domains and all motor variables. Motor variables significantly correlated with the MoCA were used in a multiple linear regression to determine the predictor variables [34, 35]. All data were expressed as mean ± standard deviation (SD), mode, median, or frequency and percentage, as required for each data type. The significance level was a p-value ˂0.05 with a 95% confidence interval (CI). |
| --- | --- | --- | --- | --- |
| Statistical methods | 12 | (*a*) Describe all statistical methods, including those used to control for confounding | 6 | All statistical analyses were performed using SPSS (v23, IBM Statistics, Armonk, NY). Participant characteristics were presented by descriptive analysis. A normality test was conducted on all variables to select the appropriate (parametric or non-parametric) statistical test. Pearson’s correlation coefficient analysis was used to assess the strength and direction of the association between cognition, muscle strength of knee extension, and motor coordination of upper extremity variables. The Spearman correlation coefficient was computed to assess associations between cognition, muscle strength of the hand grip, motor coordination of lower extremity balance, gait, and risk of fall. In addition, the Spearman correlation coefficient was used to assess the correlation between the MoCA domains and all motor variables. Motor variables significantly correlated with the MoCA were used in a multiple linear regression to determine the predictor variables [34, 35]. All data were expressed as mean ± standard deviation (SD), mode, median, or frequency and percentage, as required for each data type. The significance level was a p-value ˂0.05 with a 95% confidence interval (CI). |
|  |  | (*b*) Describe any methods used to examine subgroups and interactions | NA | NA |
|  |  | (*c*) Explain how missing data were addressed | NA | NA |
|  |  | (*d*) *Cohort study*—If applicable, explain how loss to follow-up was addressed  *Case-control study*—If applicable, explain how matching of cases and controls was addressed  *Cross-sectional study*—If applicable, describe analytical methods taking account of sampling strategy | 6 | All statistical analyses were performed using SPSS (v23, IBM Statistics, Armonk, NY). Participant characteristics were presented by descriptive analysis. A normality test was conducted on all variables to select the appropriate (parametric or non-parametric) statistical test. Pearson’s correlation coefficient analysis was used to assess the strength and direction of the association between cognition, muscle strength of knee extension, and motor coordination of upper extremity variables. The Spearman correlation coefficient was computed to assess associations between cognition, muscle strength of the hand grip, motor coordination of lower extremity balance, gait, and risk of fall. In addition, the Spearman correlation coefficient was used to assess the correlation between the MoCA domains and all motor variables. Motor variables significantly correlated with the MoCA were used in a multiple linear regression to determine the predictor variables [34, 35]. All data were expressed as mean ± standard deviation (SD), mode, median, or frequency and percentage, as required for each data type. The significance level was a p-value ˂0.05 with a 95% confidence interval (CI). |
|  |  | (*e*) Describe any sensitivity analyses |  |  |
| Results | | | | |
| Participants | 13* | (a) Report numbers of individuals at each stage of study—eg numbers potentially eligible, examined for eligibility, confirmed eligible, included in the study, completing follow-up, and analysed | 7 | Seventy subjects were enrolled, 55 females and 15 males, with a mean age of 36.60 ± 9.24 years. The participants’ demographic characteristics are presented in **Table 1.** |
|  |  | (b) Give reasons for non-participation at each stage | NA | NA |
|  |  | (c) Consider use of a flow diagram | NA | NA |
| Descriptive data | 14* | (a) Give characteristics of study participants (eg demographic, clinical, social) and information on exposures and potential confounders | 7 | Table 1 |
|  |  | (b) Indicate number of participants with missing data for each variable of interest | NA | NA |
|  |  | (c) *Cohort study*—Summarise follow-up time (eg, average and total amount) | NA | NA |
| Outcome data | 15* | *Cohort study*—Report numbers of outcome events or summary measures over time | NA | NA |
|  |  | *Case-control study—*Report numbers in each exposure category, or summary measures of exposure | NA | NA |
|  |  | *Cross-sectional study—*Report numbers of outcome events or summary measures | 7-8 | Table 2 |
| Main results | 16 | (*a*) Give unadjusted estimates and, if applicable, confounder-adjusted estimates and their precision (eg, 95% confidence interval). Make clear which confounders were adjusted for and why they were included | 8-11 | Results Pages 8-11 and Tables 3 and 4. |
|  |  | (*b*) Report category boundaries when continuous variables were categorized | NA | NA |
|  |  | (*c*) If relevant, consider translating estimates of relative risk into absolute risk for a meaningful time period | NA | NA |

Continued on next page

| Other analyses | 17 | Report other analyses done—eg analyses of subgroups and interactions, and sensitivity analyses | NA | NA |
| --- | --- | --- | --- | --- |
| Discussion | | | | |
| Key results | 18 | Summarise key results with reference to study objectives | 11-12 | his is the first study to examine the association between CI and MD in patients with MS. The analysis showed that CI is significantly associated with motor incoordination of the upper and lower extremities, balance deficits, gait abnormalities, and fall risk. However, there was no significant association between cognition and muscle strength, as estimated by hand grip and knee extension strength. Moreover, reduced upper and lower extremity coordination scores and lower fall risk were associated with higher CI scores. Specifically, the fall risk and upper extremity incoordination scores were predictive of CI in MS patients.  The results showed that the mean total MoCA score indicated mild cognitive impairment and in about two thirds of patients with CI, consistent with previous studies [38, 39]. Moreover, several studies have shown that the most affected cognitive domains in MS patients were memory, abstract/conceptual reasoning, information processing, attention, and visuospatial skills [20, 40]. Similarly, we found that memory, visuospatial skills, executive function, attention, and language were affected in patients with CI based on the MoCA scale.  This study found no association between isometric knee extensor strength and CI. Conversely, a recent study reported a significant correlation between CI and knee extension strength in MS patients [35]. Sandroff et al. [41] reported that the peak torque of knee extension was associated with cognitive processing speed but not verbal and visuospatial learning and memory in 62 people with MS. These contradictory results might be due smaller sample sizes or the type of dynamometer used. Similar to other studies, there was no correlation between hand grip strength and CI as measured by the total MoCA score [35], but there was a significant correlation between hand grip strength and the language and attention domains.  The current study revealed significant correlations between motor incoordination of the upper and lower extremities and CI in patients with MS. Poor performance on rapid alternating movement and heel-to-knee tests is usually due to cerebellar dysfunction [42]. Furthermore, several studies have shown that the cerebellum plays an important role in both the cognitive and motor functions of MS patients [42-44]. This might be because the cerebello-cerebral network, consisting of the forward cortico-ponto-cerebellar pathway and the backward cerebello-thalamo-cortical pathway, also modulates cognition [45, 46].  A balance component of POMA was significantly moderately correlated with cognitive performance in MS patients. This is the first study examining the association between cognition and balance in patients with MS using the POMA-B scale. However, other studies have examined postural-cognitive interference by incorporating the dual-task paradigms in healthy people and those with MS [47, 48]. A recent review concluded that patients with MS showed impaired balance when they simultaneously performed a cognitive and postural task, which increased their fall risk in most daily activities [47].  The gait component of the POMA was moderately positively correlated with cognition, consistent with previous studies demonstrating a significant association between gait abnormalities and cognitive decline in people with MS [49, 50]. A previous study reported that step length and step time variabilities were associated with cognitive processing speed in MS patients [50]. It is interesting to note that no study used POMA-G for the assessment of gait and its correlation with cognition in MS individuals. The current study is therefore unique in its use of a simple, short, inexpensive scale that does not need specialist equipment in clinical practice.  Fall risk was significantly correlated with cognitive impairment and predicted CI in MS individuals, consistent with previous studies [51, 52]. Moreover, a higher POMA score was associated with a decrease in fall risk, which was associated with higher MoCA scores. This is particularly important when investigating the fall risk during motor examination in patients with MS and provides a clue about the presence of cognitive impairment. Furthermore, fall frequency has been shown to be significantly correlated with general intelligence, speed of cognitive processing, and executive functioning, while verbal memory was found to be a significant predictor of falls in 81 patients with MS [52]. |
| Limitations | 19 | Discuss limitations of the study, taking into account sources of potential bias or imprecision. Discuss both direction and magnitude of any potential bias | 13 | This study has several limitations. The sample size was small, and further studies are required with a larger sample size to improve the robustness of regression. Most of the participants had relapsing-remitting MS, and future studies should involve all MS subtypes. The POMA-G used in this study is based on the examiner’s observations and is less sensitive for detecting gait abnormalities than other instruments. Therefore, future studies using advanced gait analysis systems are now needed to better detect temporal and visuospatial parameters. |
| Interpretation | 20 | Give a cautious overall interpretation of results considering objectives, limitations, multiplicity of analyses, results from similar studies, and other relevant evidence | 14 | CI is significantly associated with motor incoordination, poor balance, gait abnormalities, and increased fall risk. The fall risk and upper extremity incoordination were the best indicators of CI in patients with MS. Thus, motor assessment can provide physical therapists with clues about the presence of CI in patients with MS. In addition, incorporating coordination and balance training into the rehabilitation program may enhance cognitive functions in patients with MS, although this requires empirical testing. |
| Generalisability | 21 | Discuss the generalisability (external validity) of the study results | 13 | Most of the participants had relapsing-remitting MS which limiting the generalizability of the results on the whole MS population and future studies should involve all MS subtypes. |
| Other information | |  | | |
| Funding | 22 | Give the source of funding and the role of the funders for the present study and, if applicable, for the original study on which the present article is based | 16 | This study was not formally funded. |

*Give information separately for cases and controls in case-control studies and, if applicable, for exposed and unexposed groups in cohort and cross-sectional studies.

**Note:** An Explanation and Elaboration article discusses each checklist item and gives methodological background and published examples of transparent reporting. The STROBE checklist is best used in conjunction with this article (freely available on the Web sites of PLoS Medicine at http://www.plosmedicine.org/, Annals of Internal Medicine at http://www.annals.org/, and Epidemiology at http://www.epidem.com/). Information on the STROBE Initiative is available at www.strobe-statement.org.
